# Breast Implant–Associated CD30 Negative Peripheral T-Cell Lymphoma, NOS

**DOI:** 10.1097/HS9.0000000000000507

**Published:** 2020-12-09

**Authors:** Satish Maharaj, Drew Murray, Mohamed Hegazi, Simone Chang

**Affiliations:** Department of Hematology & Oncology, University of Louisville, Kentucky, USA.

Breast implant–associated peripheral T-cell lymphoma (BIA-PTCL) was first reported in 1997.^1^ Since then 2 clinical variants emerged, PTCL presenting as a periprosthetic seroma or a more aggressive infiltrative mass.^2-4^ After 2 decades, this PTCL received provisional classification as breast implant–associated anaplastic lymphoma kinase (ALK)–negative anaplastic large cell lymphoma (BIA-ALCL).^5^ Diagnosis is based on cytology, evidence of T-cell clonality, immunohistochemistry (IHC) for CD30 and T-cell markers, and ALK negativity.^3^ While T-cell phenotype varies, cases have been consistently CD30 positive. Consequently, CD30 has been advocated as a screening test to differentiate benign seromas. Here, we report a case of BIA-PTCL, not otherwise specified (NOS) presenting as a periprosthetic mass with no CD30 expression that was fatal. While resembling the infiltrative variant of BIA-ALCL, cell surface markers were most similar to that of monomorphic epitheliotropic T-cell lymphoma (MEITL). While this case is atypical, it suggests that even with CD30 negativity, clinicians should maintain a high index of suspicion in patients with concerning clinical or pathologic features, and BIA-PTCL, NOS with a silent phenotype should be considered. Further research into implant-associated PTCL is needed.

A 67-year-old Hispanic-American female presented to the emergency room after being found wandering in the street, disoriented. She described gradual loss of vision and could only appreciate light perception and finger movements. When her vision worsened, she saw an optometrist and started prednisone, now at 20 mg oral daily, without relief. History was remarkable for bilateral breast implant placement 42 years prior for augmentation. She denied smoking or alcohol and was a retired clerical worker. Review of systems noted significant weight loss and left breast swelling. On examination, there was a large breast mass lateral to the prosthesis with induration extending medially.

Peripheral blood testing showed normal cell counts and normal immunophenotyping by flow cytometry. Positron emission tomography-computed tomograph imaging confirmed a markedly fluorodeoxyglucose avid, dominant 10.6 × 6.7 × 12.3 cm mass within the left breast adjacent to the left breast implant (Figure). No definite hypermetabolic foci were seen outside of the dominant breast mass. However, fundoscopy showed dense vitritis and deposits in the right eye. B-scan ultrasonography confirmed posterior hyaloid separation and vitreous opacities, without retinal detachment.

**Figure.**
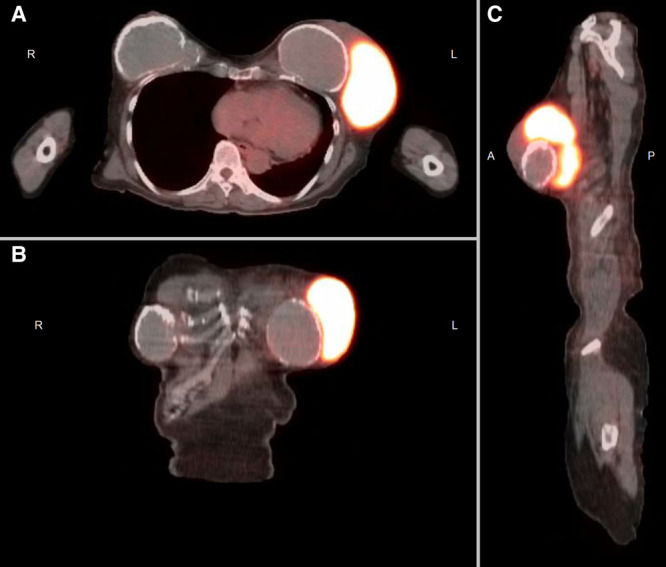
**PET-CT imaging demonstrating intensely avid periprosthetic lymphoma mass in the transverse (A), coronal (B) and sagittal (C) planes.** PET-CT = positron emission tomography-computed tomograph.

The patient underwent core biopsy of the left breast mass with extensive testing done both in house as well as at a commercial and reference facility. A summary of findings is reported in the Table. Histology showed a diffuse lymphoid infiltrate composed of large atypical cells with irregular nuclear contours, fine chromatin, conspicuous mitotic activity, and abundant eosinophilic cytoplasm. IHC was done for CD20, CD3, CD5, CD2, CD7, CD4, CD8, CD10, CD56, CD138, CD117, CD123, CD30, CD68, CD45, CD33, CD34, kappa, lambda, IgD, IgG, IgM, cyclin D1, BCL-6, BCL-2, MUM1, S-100, granzyme B, perforin, ALK1, c-MYC, PAX5, HHV8, TdT, Ki-67, pan-cytokeratin, EBER ISH, TIA-1, TCR delta, Beta F1, and ALK. The neoplastic cells were positive for CD2, CD3, CD56, Granzyme B, Perforin, c-MYC, BCL-2, MUM1, and CD20 (weak), while the rest were noncontributory. Ki-67 proliferation index was high (~90%).

**Table 1:** Pathological and Molecular Features.

Pathological Features	Phenotypical Features	EBV Status	Cytogenetic and Molecular Features
Diffuse lymphoid infiltrate composed of large atypical cells with irregular nuclear contours, fine chromatin, conspicuous mitotic activity, and abundant eosinophilic cytoplasm	Positive: Surface markers: CD2, CD3, CD56, Granzyme B, Perforin, c-MYC, BCL-2, MUM1, and CD20 (weak); Ki67 proliferative index >90% Negative: Surface markers: CD5, CD7, CD4, CD8, CD10, CD14, CD19, CD23, CD138, CD117, CD123, CD30, CD68, CD45, CD33, CD34, CD79b, Kappa, Lambda, IgD, IgG, IgM, Cyclin D1, BCL-6, S-100, ALK1, PAX-5, HHV8, TdT, Ki-67, pan-cytokeratin, TIA-1, TCR delta, Beta F1, and ALK. Intracytoplasmic markers: Tdt, MPO, CD22, CD3, CD79a	EBER negative	Positive: T-cell receptor Gamma gene rearrangement, BCL2 deletion or chromosome 18 monosomy Negative: BCL6 rearrangement, MYC rearrangement/amplification, IgH/BCL2 translocation

Fluorescence in situ hybridization studies were negative for BCL-6 rearrangement, MYC rearrangement, MYC amplification, or IgH/BCL-2 translocation. The interphase study revealed a deletion signal pattern in the 14;18 (IgH/BCL2) probe set suggestive of a BCL2 gene deletion or chromosome 18 monosomy. Polymerase chain reaction studies were positive for T-cell receptor gamma gene rearrangement and negative for B-cell gene rearrangement, confirming a clonal T-cell population. Sampling of both vitreous and cerebrospinal fluids was performed and flow cytometry confirmed central nervous system (CNS) infiltration, with distinct populations ranging from 18% to 37% cellularity exhibiting a cytotoxic phenotype and falling within the high forward scatter region indicating large size.

The patient was diagnosed with BIA-PTCL, NOS with CNS involvement. After an extensive discussion on treatment options, she decided on comfort-directed measures and was discharged in the care of her family with hospice care; she died 4 weeks later.

Primary non-Hodgkin lymphomas of the breast constitute 0.01%-0.5% of all malignant breast tumors and 1% of all non-Hodgkin lymphoma.^6,7^ Breast PTCL is even more infrequent, representing 8%-10% of all breast lymphomas.^3^ With the first BIA-PTCL case reported in 1997, the next 2 decades saw increasing reports, and in 2016, provisional classification for BIA-ALCL was published.^5^ The exact incidence of BIA-ALCL is unknown but it is estimated that the lifetime prevalence is 1 per 30,000 patients with textured breast implants, although a prospective study suggested that the incidence may be closer to 1 case per 4000.^8^ Most cases present after 7-10 years and at a median age of 50-60 years,^3,9^ although there are reports >30 years from surgery, as in the present case.^10^

Our current understanding is that there are 2 clinical variants of BIA-ALCL, presenting as either the more common periprosthetic seroma or an infiltrative mass.^2-4^ These 2 variants have different prognoses, with the infiltrative variant having more aggressive biology and worse outcomes. However, no specific pathologic features differentiating these 2 groups have been elucidated.^3,11^ Indeed, it is unclear if these are distinct subtypes, or simply represent a spectrum of disease.

BIA-ALCL is thought to develop after years of implant-induced inflammation inducing dysplastic changes from chronic antigen stimulation. To date, the 2 most frequently discussed etiological candidates are inflammation from textured implants and chronic bacterial stimulation.^12^ Added to this is a recent hypothesis on prosthesis-associated tissue hypoxia.^11^ Aberrant gene expression is of course most likely involved in the oncogenic progression from a chronic inflammatory state to a full-blown malignancy. Recurrent JAK1 and STAT3 gene mutations have been identified and, like many other ALCLs, also identified have been mutations in DNMT3A, TP53, and SOCS.^13-15^ However, even in the analyses of these small series, there have been cases evaluated with no somatic mutations. Recently, RNA sequencing and gene set enrichment analysis comparing eleven BIA-ALCLs to 24 non–BIA-ALCLs identified upregulation of hypoxia signaling genes including VEGFA, VEGFB, SLC2A3 (encoding GLUT3), and carbonic anyhydrase-9.^11^

Currently, diagnosis of BIA-ALCL is based on cytology, evidence of T-cell clonality, and IHC. Morphologically, the tumor is comprised of large, pleomorphic cells with abundant cytoplasm, and horseshoe-shaped or “embryoid” nuclei with prominent nucleoli. With the exception of prominent nucleoli, the case presented had this morphology, and as noted, also T-cell clonality. On IHC, BIA-ALCL typically demonstrates strong and uniform membranous expression of CD30 and lacks ALK expression. Other T-cell antigens have been reported expressed variably, with the most common being CD4 (79%-84%), CD43 (80%-95%), CD3 (28%-46%), CD45 (36%), and CD2 (30%-41%).^3,10,16^ Expression of CD5, CD7, CD8, CD15, or CD56 is less common. MUM1, Granzyme B, TIA-1, and BCL2 have a high prevalence.^3,10,16^ Our case expressed CD2, CD3, CD56, Granzyme B, Perforin, C-MYC, BCL-2, MUM1, and CD20 (weak). Weak expression of CD20 has been reported in PTCL, NOS.

While T-cell phenotype has been variably reported, cases to date have been consistently CD30 positive. For instance, in a 2019 expanded safety analysis, 246 cases reported positive.^2^ Nevertheless, IHC has not been reported in all cases, and for example, in this series, 57% of cases did not have CD30 results available.^2^ National Comprehensive Cancer Network guidelines now recommend that scant or rare CD30-positive lymphocytes with normal morphology can be considered a normal finding with no further investigation.^17^ One group has recently examined a CD30 enzyme-linked immunosorbent assay with the aim of a lower-cost screening test more widely available than flow cytometry and IHC, and use to screen suspicious peri-implant fluid collections.^18^ Other groups are investigating unique cytokine profiles including IL-13, and molecular signatures to differentiate BIA-ALCL.^11,15,19^ Apart from the significance of CD30 in diagnosis, it is also important for management. Brentuximab vedotin has been used in cases of BIA-ALCL.

This case of BIA-PTCL, NOS presented a large periprosthetic mass with the dilemma of no CD30 expression. The disease was extremely aggressive with a high proliferation index and CNS involvement, and ultimately resulted in death. Clinically, the disease course resembled the infiltrative type of BIA-ALCL. Cytology, ALK testing, and T-cell clonality were similar to reported cases of BIA-ALCL. However, the cell surface markers were most similar to that of MEITL. Both MEITL and BIA-ALCL are novel diseases classified in 2016 and driven by T-cell activation. MEITL was previously known as type II enteropathy–associated T-cell lymphoma but it was given a new name due to its distinctive nature and lack of association with celiac disease. It should be noted that MEITL is an intestinal lymphoma and does not occur in the setting of implants. In this case, it could easily be excluded based on the clinical setting, tumor site, as well as histology—MEITL tumor cells are monomorphic and small to medium in size, infiltrating the intestinal epithelium. The adjacent “normal” mucosa demonstrates villous atrophy, crypt hyperplasia, and intraepithelial lymphocytosis. There is no inflammatory background in MEITL. Most cases of MEITL are derived from γδ T cells and, similar to BIA-ALCL, the JAK-STAT pathway has been implicated in MEITL.^5,20^

While this case is rare, it suggests that even with CD30 negativity, clinicians should maintain a high index of suspicion in patients with concerning clinical or pathologic features, and consider BIA-PTCL, NOS with silent phenotype. As more cases of BIA-ALCL are reported, we anticipate more data on atypical cases. Further research into BIA-PTCL, NOS is needed.

We have presented the first case of BIA-PTCL, NOS occurring as CD30 negative disease with a surface phenotype similar to MEITL. The disease was clinically similar to the infiltrative mass variant of BIA-ALCL, being aggressive and resulting in death. This case suggests that CD30 by itself is not 100% sensitive to exclude BIA-PTCL, NOS. Similar cases need to be reported to help define this disease better and further research into implant-associated PTCL in general is urgently needed.
